# Study on Mechanism of Structure Angle on Microstructure and Properties of SLM-Fabricated 316L Stainless Steel

**DOI:** 10.3389/fbioe.2021.778332

**Published:** 2021-11-03

**Authors:** Xiaofeng Li, Denghao Yi, Xiaoyu Wu, Jinfang Zhang, Xiaohui Yang, Zixuan Zhao, Jianhong Wang, Bin Liu, Peikang Bai

**Affiliations:** ^1^ School of Materials Science and Engineering, North University of China, Taiyuan, China; ^2^ The State Key Laboratory of Powder Metallurgy, Central South University, Changsha, China; ^3^ Institute of Laser Engineering, Faculty of Materials and Manufacturing, Beijing University of Technology, Beijing, China; ^4^ Instrumental Analysis Center, Taiyuan University of Science and Technology, Taiyuan, China

**Keywords:** selective laser melting, construction angles, 316L SS, tensile properties, grain refinement

## Abstract

In this study, seven 316L stainless steel (316L SS) bulks with different angles (0°, 15°, 30°, 45°, 60°, 75°, and 90°) relative to a build substrate were built *via* selective laser melting (SLM). The influences of different angles on the metallography, microstructure evolution, tensile properties, and corrosion resistance of 316L SS were studied. The 0° sample showed the morphology of corrugated columnar grains, while the 90° sample exhibited equiaxed grains but with a strong <101> texture. The 60° sample had a good strength and plasticity: the tensile strength with 708 MPa, the yield strength with 588 MPa, and the elongation with 54.51%. The dislocation strengthening and grain refinement play a vital role in the mechanical properties for different anisotropy of the SLM-fabricated 316L SS. The 90° sample had greater toughness and corrosion resistance, owing to the higher volume fraction of low-angle grain boundaries and finer grains.

## Introduction

Additive manufacturing (AM) has great development prospects in the medical, automotive, aerospace, and mold industries and is currently a global manufacturing trend, as stated by Song S. et al. (2020). Selective laser melting (SLM), as one of the AM technology, “prints” materials and components directly from a computer-aided design file, thereby offering unique advantages of design freedom for complex parts without the need for molds (Shu et al., 2020). As SLM is a layer-by-layer building technology, it provides ample opportunities for tailoring the microstructure and then mechanical properties (Xing et al., 2020). At present, the powder materials suitable for SLM technology mainly include 316L stainless steel, AlSi10Mg, Inconel 718, Inconel 625, and TC4 (Harun et al., 2018; Kreitcberg et al., 2017; Li et al., 2021; Luo et al., 2019; Yu et al., 2020).

Notably, 316L stainless steel (316L SS) has excellent comprehensive mechanical properties, corrosion resistance, good biocompatibility, and good SLMed formability (Kong et al., 2018a; Shin et al., 2021; Xu et al., 2021), but the release of Ni element may cause adverse health problems. Therefore, the important factor for the application of 316L SS in the biological field is the corrosion and element release of the SLM-formed part in the biological environment. The SLM-processed 316L SS had been extensively studied, including relative density, surface roughness, microstructure, and mechanical properties (Liverani et al., 2017; Agrawal et al., 2020; Marattukalam et al., 2020). However, the SLM-fabricated 316L SS displays microstructure anisotropy. Specifically, the difference of columnar grains of the vertical plane and equiaxed grains of the horizontal plane could cause anisotropy of mechanical properties and corrosion resistance. Additionally, defect distribution, residual stress, deformation mechanism, and melt pool boundary are affected by the performance anisotropy of the SLM-prepared 316L SS (Yin et al., 2018; Chen et al., 2019; Shifeng et al., 2014; Blinn et al., 2020). In order to alleviate this, some researchers have decreased defects in the SLM-processed 316L SS, such as cracks and pores, by optimizing the process parameters (Liverani et al., 2017). Several studies have changed the laser-scanning strategy and increased the preheating temperature to improve the residual stress level and the microstructure anisotropy within the SLM-fabricated 316L SS (Song Y.n. et al., 2020). AlMangour et al. (2017) have added nano-scale TiC particles to improve the SLM-processed 316L SS’ mechanical performances by refining the microstructure and increasing dislocation density. Additionally, many researchers have implemented post-processing techniques, such as heat treatment, promoting the homogenization of microstructures and improving the mechanical performances anisotropy from the SLM-formed 316L SS (Hong et al., 2021). Kong et al. (2018a) found that 316L SS formed under high laser power has a thicker passivation film and higher cell proliferation ability than that formed at low laser power.

It is crucial to study the influence of the construction angle on the SLM-prepared 316L SS’ microstructure evolution, mechanical performances, and corrosion under stable process parameters. During SLM, the relationship between the placement of the sample and the gas–flow direction can affect the relative density of the formed part (Agius et al., 2017). Build orientation, local heat input, and rapid solidification can change the grain orientation of SLM-prepared 316L SS. Additionally, Wen et al. (2019) found that the spatial orientation of the molten pool boundary affected the SLM-fabricated 316L SS’ toughness. The fatigue–crack direction of SLM-fabricated 316L SS was correlated with the laser scanning angle, and the formation of defects impacts the type of fractures (Zhang et al., 2019). Liu et al. (2020) reported that the crystal plasticity model accurately captured and forecasted the yield strength anisotropy behavior of SLM-fabricated 316L, which is mainly influenced by the crystallographic structure. During deformation, the vertical sample was deformed by grain extension then dislocation slip and had the stability of work hardening, whereas grain rotation occurred through slipping and massive twinning in the horizontal sample, which exhibited a smaller effective grain size (Bahl et al., 2019; Liu et al., 2020; Kale et al., 2021). Thus, the 0° sample had higher yield strength than the 90° sample. Atapour et al. (2020) studied the corrosion resistance of SLM-formed 316L SS from different build orientations before and after heat treatment in dilute hydrochloric acid (HCl, PH = 1.5). Compared with as-built, the heat-treated sample could release more Ni element, which may be attributed to the reduction of the surface oxide layer thickness.

In this study, seven groups of 316L SS samples with different angles relative to a build substrate were designed and then formed through SLM technology using optimized process parameters. The effects of construction angles (from 0° to 90° relative to build plate) on the microstructure evolution, texture, tensile properties, and corrosion resistance of SLM-prepared 316L samples were researched. This study drew an outlook on the gradient in microstructure evolution, tensile properties, and corrosion resistance from different construction angles relative to the build substrate in SLM-fabricated 316L SS.

## Experiment

### SLM Technique

**FIGURE 1:**
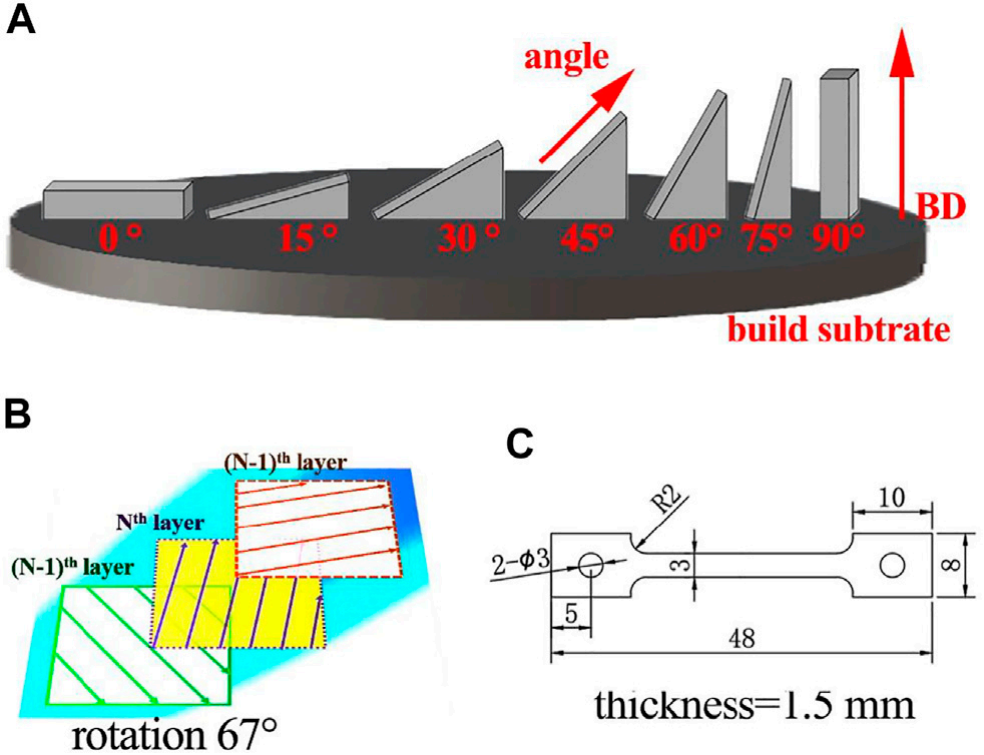
Model design: **(A)** 45° sample, **(B)** 75° sample, **(C)** selected laser scanning strategy, and **(D)** dimensions (in mm) used in the fabrication of the tensile samples.

A gas-atomized 316L stainless steel powder with a particle size ranging from 20 to 50 μm was produced by Avimetal Powder Metallurgy Technology Co., Ltd. The SLM samples were prepared employing the Renishaw AM 400 system. The beam size of laser was 70 μm in diameter. The SLM processes were proceeded in high-purity Ar (≥99.99%) gas atmosphere with an O_2_ content below 0.1%. Optimized printing parameters of 50-μm powder layer thickness, 185-W laser power, 100-μs exposure time, and 120-μm hatch distance were used to ensure good material density. The selection of optimal parameters was determined on the basis of a large number of experiments. During the SLM process, a rotation of 67° laser scanning strategy between successive layers was used, as shown in Figure 1C. Seven samples with different angles relative to the build plate were manufactured. One sample was prepared every 15° from 0° to 90°. Figure 1A,B shows the models of the 45° sample and 75° sample. There was no dangling part in the SLM-processed sample, and the deposition area of the sample (construction angle from 0° to 90°) gradually decreased with the increase in the built height. Among them, the 0° sample had the largest deposition area (55 × 10 mm), and the 90° sample had the largest *Z*-axis dimension (55 mm).

### Microstructure Characterization and Mechanical Properties

To experimentally study the mechanical anisotropy, tensile samples were fabricated along different construction angles relative to the build subtrate: 0°, 15°, 30°, 45°, 60°, 75°, and 90°. The selection of the construction angles was conducive to a more comprehensive understanding of the force on each part of the SLM-fabricated sample. Blocks close to the net dimension were cut out of each sample *via* electric discharge machining (EDM) and used for producing tensile samples. Figure 1D shows the three-dimensional size of a tensile sample. The cut blocks were ground using SiC paper, polished by diamond suspension, and etched with an acid mixture (HCl:HNO_3_ = 3:1). Using an Axiovert 200MAT optical microscope (OM) and a TESCAN VEGA3 scanning electron microscope (SEM), we observed the macrostructure or microstructure of each samples. Electron backscatter diffraction (EBSD) results were obtained on a Hitachi S-3400N SEM equipped with an HKL EBSD. The EBSD measurements were done on extractions from the 0°, 45°, 60°, and 90° samples, and the test plane was selected to be perpendicular to the load–direction plane. Using a calibrated E43.504 electronic universal testing machine, we performed the tensile tests of each sample at room temperature, with a 2 mm/min tensile speed. Samples were tested in triplicate to ensure accuracy.

### Electrochemical Testing

The electrochemical measurements were performed in 3.5 wt% NaCl solution at 25°C, and the etching surface zone was 5 mm × 5 mm. A three-electrode arrangement was used: the sample is the working electrode, saturated calomel is the reference electrode, and a Pt is the counter electrode. All of the samples were welded by a copper wire and then embedded in the epoxy resin. The working surfaces were ground with SiC paper, polished, ultrasonically cleaned in absolute ethyl alcohol for 3 min, and air-dried before testing. The corrosion resistance of all samples was characterized by polarization curves. The corrosion current and sweep potential of each sample were analyzed by CHI604E software. Each sample’s current density is based on the total current divided by the exposed area during the corrosion test. The surface roughness is not generally considered in the calculation. The calculation can be expressed as follows:

$$i_{\mathrm{corr}}=\frac{I_{\mathrm{corr}}}{A},$$

where i_corr_ is current density with A/cm^2^, I_corr_ is total anodic current with unit of A.

## Results

### Macrostructure Evolution

**FIGURE 2:**
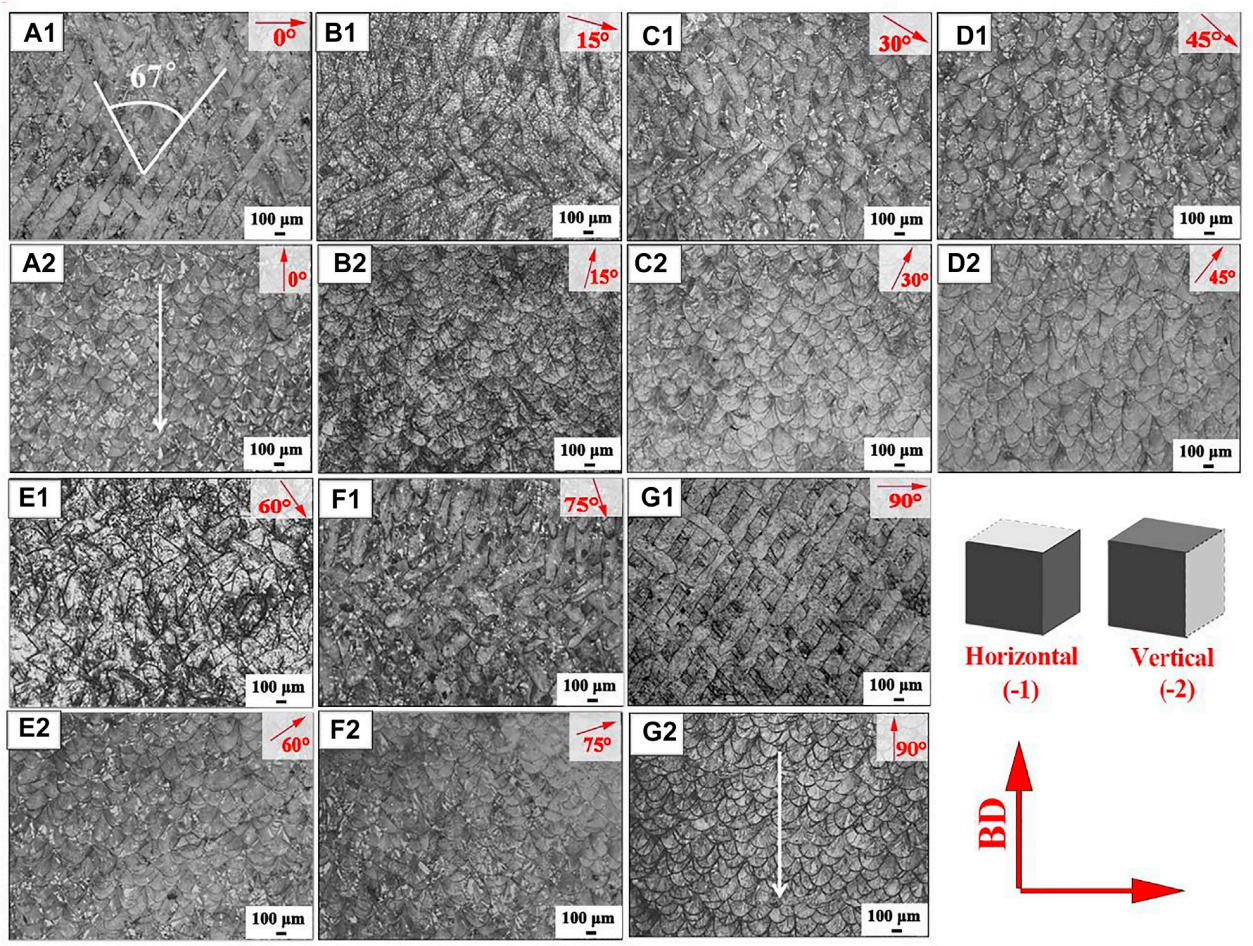
OM images of SLM-processed 316L SS from different angles relative to the build substrate: (a_1_-g_1_) cross section and (a_2_-g_2_) longitudinal section.

In Figure 2, the OM images show the morphologies form in the horizontal and vertical planes of the SLM-formed 316L SS from different angles (relative to build subtrate). Figure 2A_1_-g_1_ shows the “track–track” overlap trajectory that was the overlapping trajectory between the n^th^ laser track and the (n+1)^th^ laser track. Consistent with the scanning pattern, the laser track of samples with different construction angles displayed intersection angles of 67° (shown by the white line in Figure 2A_1_). Shortened and even overlapped average laser tracks were observed for each set of construction angle samples (for which the sum of construction angles = 90°) with the construction angle changes. The sets of construction angles were: 0° and 90° samples, 15° and 75° samples, and 30° and 60° samples. The laser track of the horizontal plane of the 45° sample showed a semicircular melt channel, similar to the shape of the molten pool.

Figure 2A_2_-g_2_ shows the microscopic morphology of the vertical planes of the SLM-formed 316L SS from different angles (relative to build subtrate). Fish scale–like melt pools were observed in the vertical plane of all samples and were repeatedly stacked to form dense SLM-processed parts. Each sample showed similar melt pool morphology, which showed a “layer–layer” overlap. The tilt angle of the melt pools between the adjacent layers also changed with the construction angle. The melt pool orientation of the 0° and 90° samples was perpendicular to the build plate, see in Figure 2A_2_ and g_2_, while a part of the melt pool orientation from the 15° sample was at an angle of 15° relative to the build plate; other samples followed similar patterns.

### Microstructure Evolution

**FIGURE 3:**
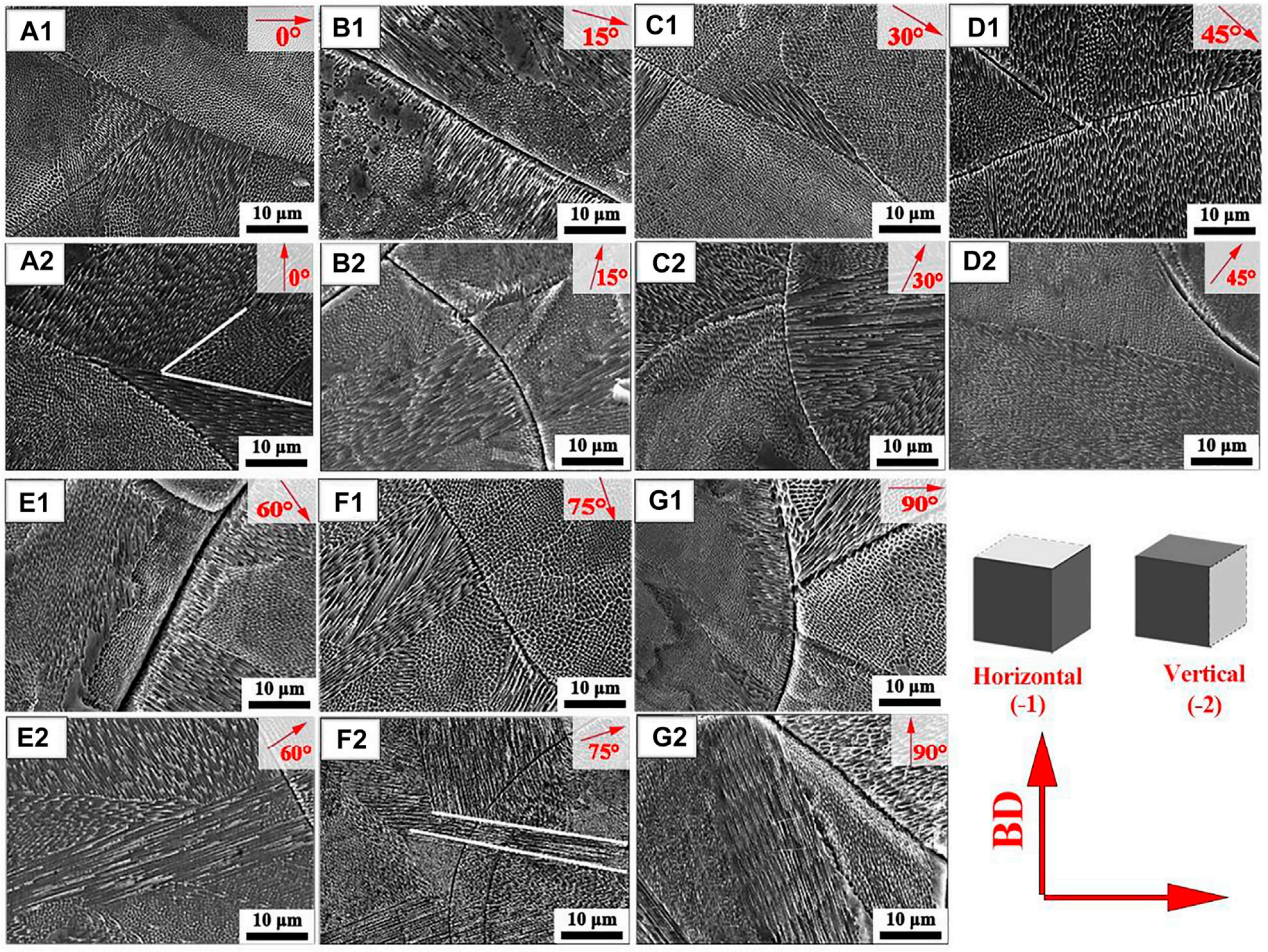
SEM images of SLM-processed 316L SS with from different angles relative to the build substrate: (a_1_-g_1_) cross section and (a_2_-g_2_) longitudinal section.

As shown in Figure 3A_1_-g_1_, the SEM morphology of the cross section from the SLM-formed 316L SS with different angles (relative to build subtrate) were exhibited. The grains of SLM-formed 316L SS were mainly composed of columnar subcrystals, cellular subcrystals, and cellular dendrite subcrystals. The 60° samples exhibited finer cellular subcrystals and cellular dendrite subcrystals, followed by the 45°, 30°, 0°, and 15° samples in the order of fineness. The 75° and 90° samples had larger cellular subcrystals and cellular dendrite subcrystals. Cellular dendrite subcrystals were observed in the 0° sample and 60° sample, while columnar subcrystals of different orientations appeared in the 30° sample, 45° sample, and 75° sample. The 15° sample and 90° sample displayed columnar subcrystals perpendicular to the molten pool boundary.

Figure 3A_2_-g_2_ exhibits the SEM morphology of the longitudinal section from the SLM-formed 316L SS with different construction angles. There were cellular subcrystals inside the melt pool of the 0° and 45° samples, and a small number of cellular dendrite subcrystals outside the melt pool boundary (MPB). The 15° sample displayed cellular dendrite sub-grains inside the melt pool. Figure 3C_2_ shows that the columnar subcrystals were perpendicular to the MPB from the 30° sample. The 60° sample and 90° sample exhibited mostly cellular dendrite subcrystals and cellular subcrystals. The cellular dendrite subcrystals of the 60° sample were of an uniform size, while the cellular dendrite subcrystals of the 90° sample were slightly larger, as shown in Figure 3E_2_ and g_2_.

Comparing the SEM images in Figure 3A_1_-g_1_, the proportion of columnar sub-grains in the longitudinal section was higher than that in the cross section, especially in the 75° and 90° samples. The proportion of dendrite sub-grains increased in order of 15°, 0°, 60°, 30°, and 45° samples. The orientation of dendrite sub-grains from 75° sample and 90° sample exhibited obvious differences, as shown in Figure 3F_2_ and g_2_. Figure 3F_2_ shows the epitaxial growth of dendrites in the 75° sample. The dendrite grains inside the melt pool continued to cross and grow along the melt pool boundary to a new melt pool, and dendrite grains of different orientations appeared.

**FIGURE 4:**
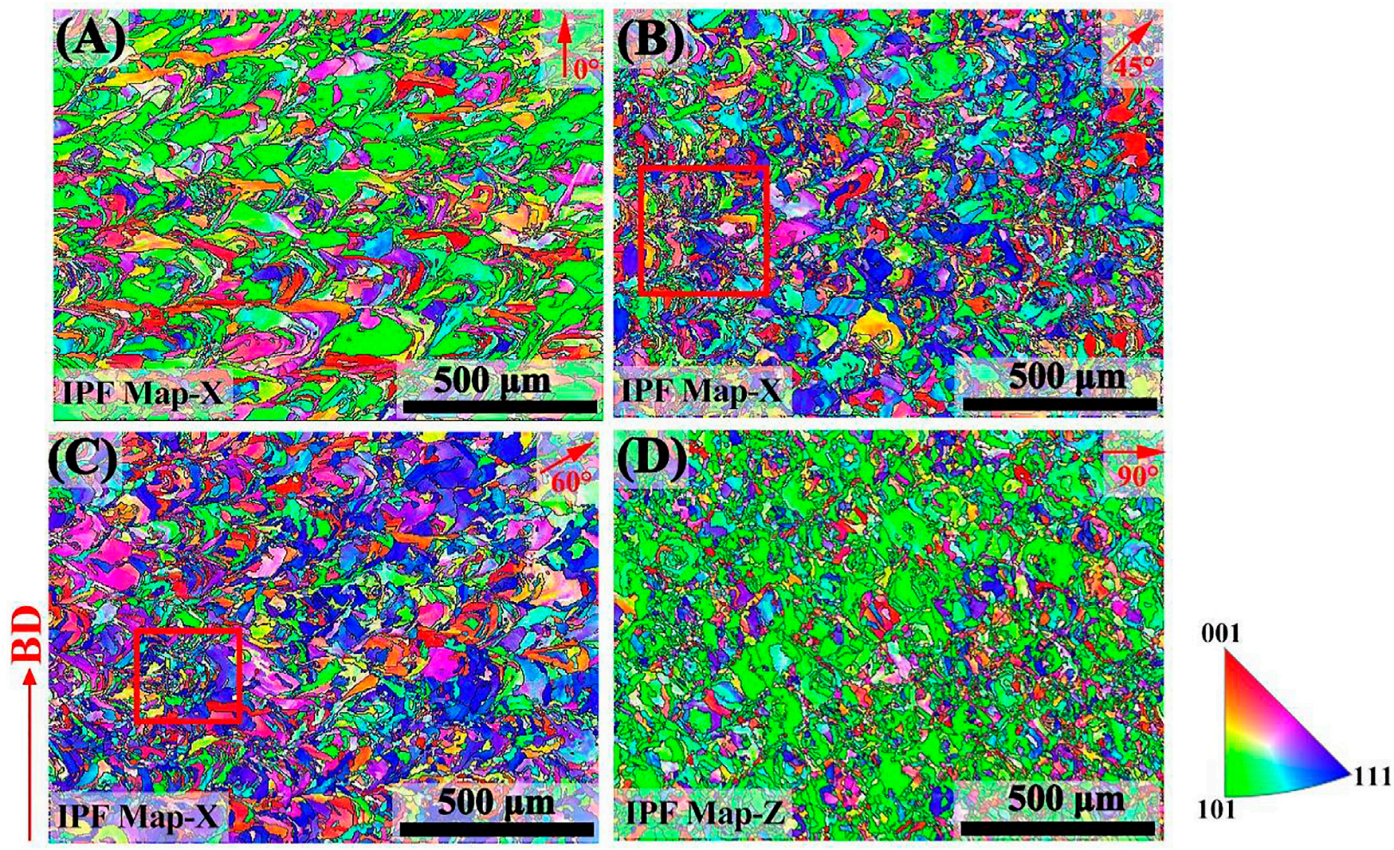
EBSD inverse pole figures of SLM-processed 316L SS from different angles relative to the build substrate: **(A–D)** angle of 0, 45, 60, and 90.

Figure 4 shows the inverse pole figures (IPF) of the sample with different angles of 0°, 45°, 60°, and 90°. The microtexture was analyzed based on the EBSD data obtained from a plane perpendicular to the load direction. The grain orientation maps were mainly identified by three color: red is <001>, green is <101>, and blue is <111> (Kale et al., 2021). No sign of MPBs appears due to epitaxial grain growth in remelted zones, shown in Figure 4A. The 0° sample’ grains show a ripple pattern and a strong <101> texture (represented by green) obtained parallel to the build direction. The 45° and 60° sample exhibited a non-uniform grain structure and numerous finer grains appeared, as shown in the red boxes in Figure 4B,C. The portion of <111> oriented grains (displayed by blue color) of the 45° sample was higher than those of the 0° and 90° samples, while the portion of <111> oriented grains of the 60° sample was higher compared with the 45° sample. The <111> oriented grains showed an increasing trend with the increase in the construction angle. The 90° sample showed rectangular beam–shaped grains, which were marked with equiaxed grains in the scanning direction, and an intense <101> texture was acquired along the scan direction. An additional, minor <001> texture component was visible in all the samples.

According to the EBSD data, the 0°, 45°, 60°, and 90° samples included 1,929, 2,980, 2,583, and 3,316 crystal grains, respectively. The average grain sizes of these samples were 25.18, 20.19, 22.49, and 19.93 μm, as measured by the interception method. The proportions of grains smaller than 11.0 μm in the 0°, 45°, 60°, and 90° samples were 5.02, 0.00, 5.13, and 5.94%, respectively. In addition, the aspect ratios of grains from the 0°, 45°, 60°, and 90° samples were 3.12, 2.45, 2.52, and 2.18, respectively. The larger the aspect ratio of the grains, the higher the proportion of columnar grains in the plane (Spittle, 2013). Therefore, the 0° sample showed a high proportion of columnar grains, followed by the 60° sample, and the 45° sample.

**FIGURE 5:**
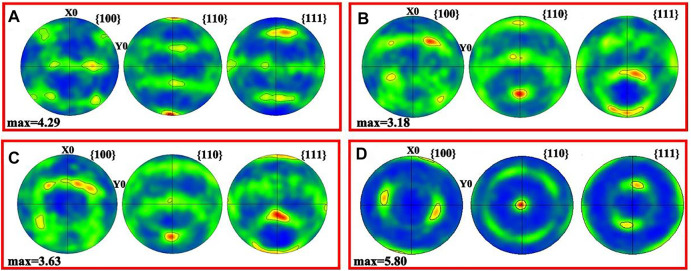
PF of SLM-processed 316L SS from different angles relative to the build substrate: **(A–D)** angle of 0, 45, 60, and 90.

Figure 5 shows the pole figures (PF) of each samples with different angles of 0°, 45°, 60°, and 90°, corresponding to the 0° sample, 45° sample, 60° sample, and 90° sample. The grains of the 0° sample were symmetrically arranged in parallel to the X0 axis, and an intense <110> crystallographic texture was acquired parallel to the BD. The 90° sample showed a preferred <110> texture perpendicular to the XY plane. The diffusion ring seen from the {110} PF demonstrated a fiber texture parallel to the X-orientation. Based on the Channel 15 analysis, the maximum texture was a shear texture of 45° with the build direction, as shown in the {110} PF of the 45° sample. Figure 5C shows that the maximum texture was a shear texture of 60° with the build direction in the {110} PF of the 60° sample. It illustrated that the grain orientation of the different angle planes was related to the build direction. The multiple of the uniform density (MUD) value depicts the relative texture strength of a particular orientation in a sample. The 90° sample showed a larger MUD value (5.80), followed by the 0° sample (MUD = 4.29). The MUD of the 60° sample was 3.63, and the 45° sample had the minimum mud value of 3.18.

**FIGURE 6:**
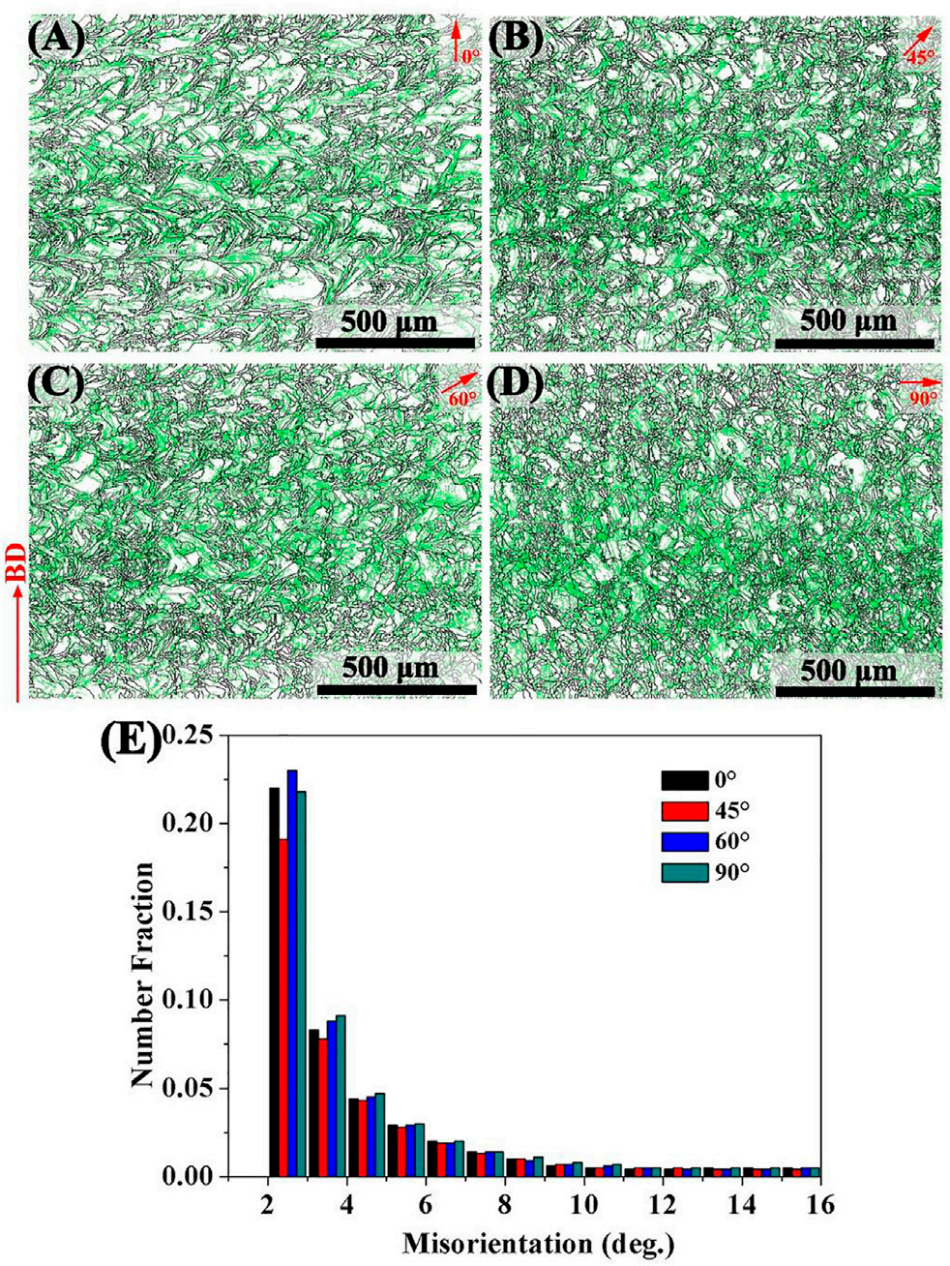
Grain boundary misorientation angles of SLM-processed 316L SS from different angles relative to the build substrate: **(A–D)** angle of 0, 45, 60, and 90 and **(E)** the proportion of LAGBs in each sample.

Figure 6 shows the grain boundary misorientation angle distribution of SLM-formed 316L SS with different angles of 0°, 45°, 60°, and 90°, corresponding to the 0° sample, 45° sample, 60° sample, and 90° sample, respectively. The green and black lines in Figure 6 correspond to the locations of LAGBs and HAGBs, respectively. Among them, grain boundaries with an orientation angle less than 15° were defined as LAGBs, while the rest were defined as HAGBs. The statistical results are summarized in Figure 6E. The linear fractions of LAGBs from the 0°, 45°, 60°, and 90° samples were 45.4, 41.6, 46.9, and 47.1%, respectively. Of them, the linear fractions of LAGBs were the lowest in the 45° sample, while the 60° and 90° samples had similar linear fractions of LAGBs.

**FIGURE 7:**
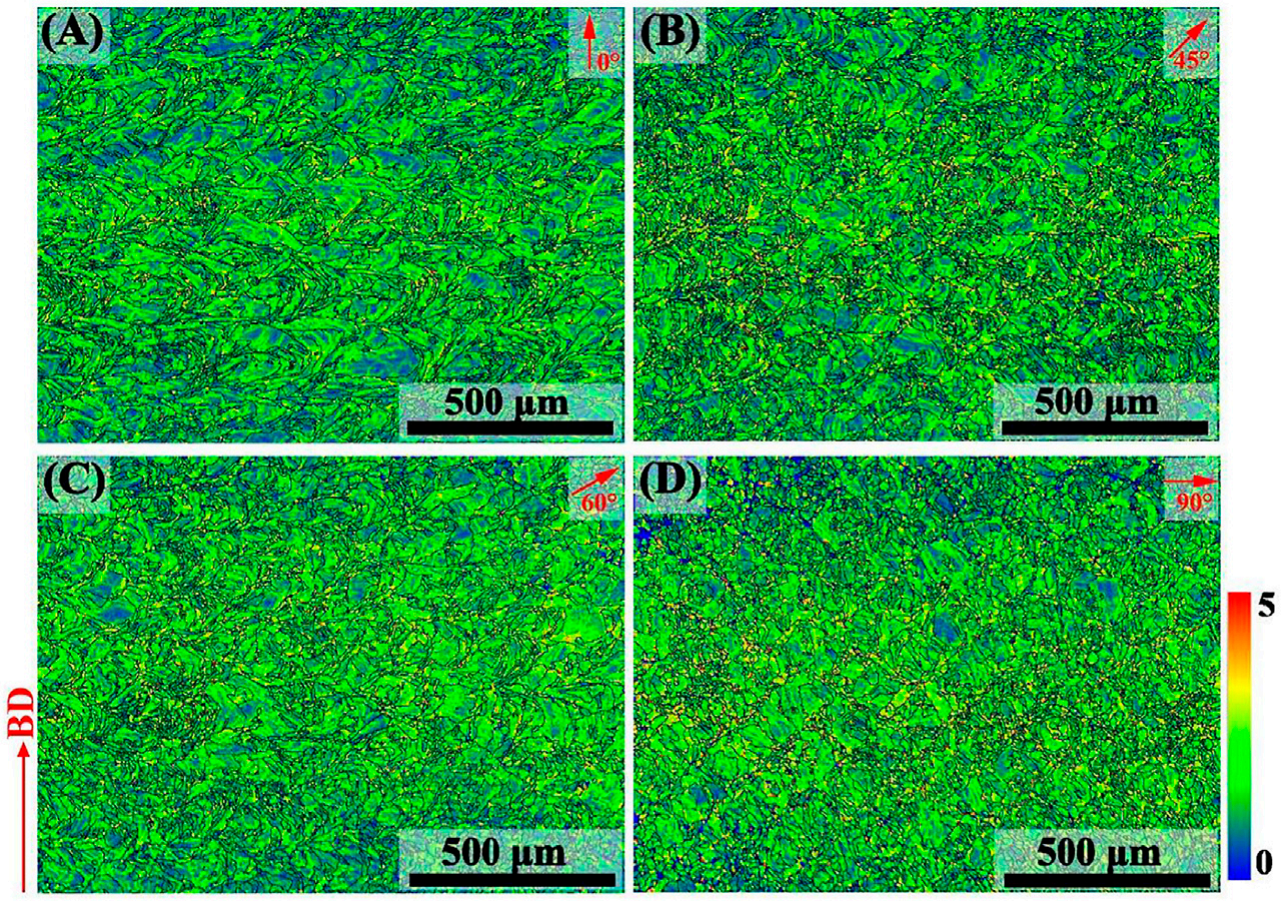
KAM map of SLM-processed 316L SS from different angles relative to the build substrate: **(A–D)** angle of 0, 45, 60, and 90.

The kernel average misorientation (KAM) diagrams represent the average misorientation of a given point relative to the third nearest neighbor (upper limit of 5°), as shown in Figure 7. The green color corresponds to a high stress concentration and high degree of plastic deformation. Based on the qualitative analysis of the KAM image, the SLM-prepared 316L SS exhibited high residual stress, which results from multiple thermal cycles and especially thermal shrinkage stress during rapid melting and solidification. To convert local misorientation into the density of geometrically necessary dislocation (GND), the following formula is used:

$$\rho^{\mathrm{GND}}=\frac{2\theta }{\mathrm{ub}},$$

where $\rho^{GND}$ is the GND density at points, θ denotes the local misorientation angle (Gao et al., 2020), b represents the Burger’s vector (0.25 nm), and u is the scan step (3 μm) of EBSD. The average $\rho^{GND}$ of the 0°, 45°, 60°, and 90° samples were 3.89 × 10^15^ m^−2^, 4.26 × 10^15^ m^−2^, 4.29 × 10^15^ m^−2^, and 4.56 × 10^15^ m^−2^, respectively.

### Tensile Performance and Fracture Examination

**FIGURE 8:**
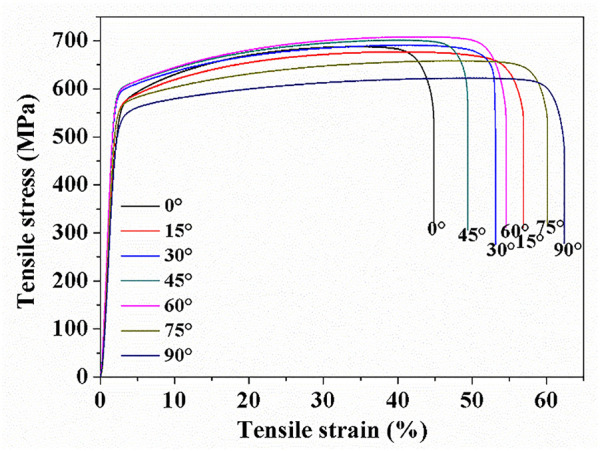
Stress–strain curve of SLM-processed 316L SS from different angles relative to the build substrate.

Figure 8 depicts the stress and strain curves of all samples, and the corresponding ultimate tensile strength (UTS), yield strength (YS), and elongation are listed in Table 1. The samples with different construction angles showed obvious mechanical anisotropy. The 45° and 60° samples exhibited the maximum tensile strengths of 701 and 708 MPa, respectively. This tensile strength was significantly better than the SLM-formed 316L SS of Song Y.n. et al. (2020) and Chao et al. (2021). The 90° sample had lower tensile strength (623 MPa) than the other samples. The 45° sample and 60° sample had approximately equal yield strength, with values of 586 and 588 MPa, respectively. The 0° and 90° specimens showed lower yield strength, with values of 538 and 513 MPa, respectively. The 90° sample displayed the best plasticity, with a maximum elongation of 62.57%. The elongation of the 45° sample (49.25%) was only slightly better than that of the 0° sample (45.21%).

**TABLE 1:** Tensile properties of SLM-processed 316L SS with different angles relative to the build substrate.

Construction angle (°)	UTS (MPa)	YS (MPa)	Elongation%
0	688	538	45.21
15	677	551	57.94
30	691	566	53.66
45	701	586	49.25
60	708	588	54.51
75	658	560	60.92
90	623	513	62.57

**FIGURE 9:**
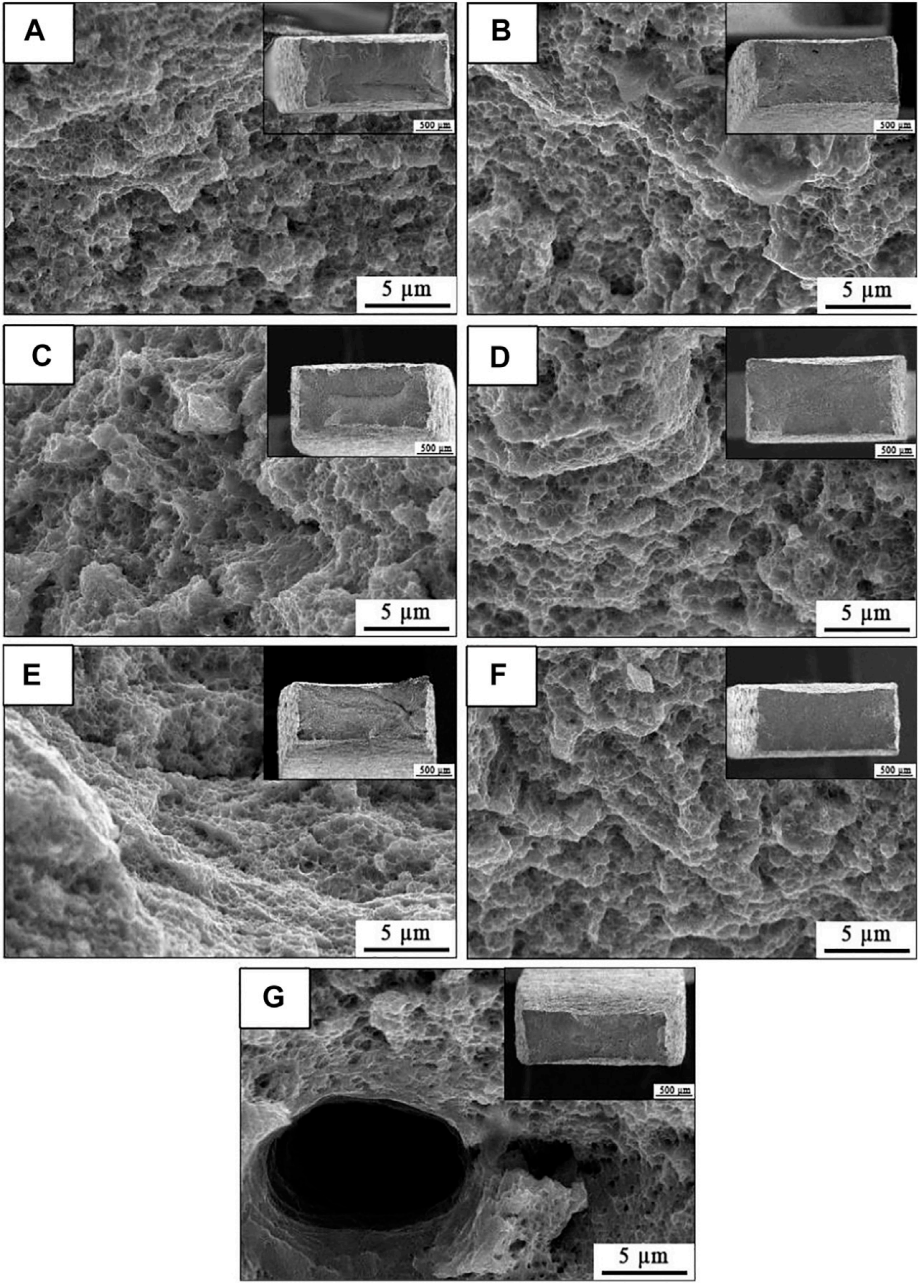
Characterization of fracture surface of the SLM-processed 316L SS from different angles relative to the build substrate: **(A–G)** angle of 0, 15, 30, 45, 60, 75, and 90.

Figure 9 displays the fracture morphologies of the SLM-formed 316L SS from different angles relative to the build substrate, and the illustrations show the corresponding macroscopic fracture morphology. It can be seen that the fractures of each sample were smooth fracture facets, showing the necking phenomenon. The 0° sample and 45° sample exhibited slight necking, while the 75° and 90° samples displayed more necking, corresponding to the stronger plasticity. The 45°, 75°, and 90° samples were relatively flat in the fracture surface, while the 0°, 30°, and 60° samples had fine cracks, which results from the necking stage causing the fast fracture of a sample. Pores were visible in the fracture plane of the 15° sample, indicating that stress preferentially accumulated at the pore. A dimple morphology was observed in every alloy, indicating that the SLM-processed 316L SS had good plasticity. The 60° sample displayed relatively smaller and uniform dimples, indicating uniformity of the microstructure. The 90° sample had large and deep dimples with a diameter of about 5 μm, indicating that the sample had a great capability to lose stability locally. Thus, the 90° sample had high plasticity.

### Corrosion Resistance

**FIGURE 10:**
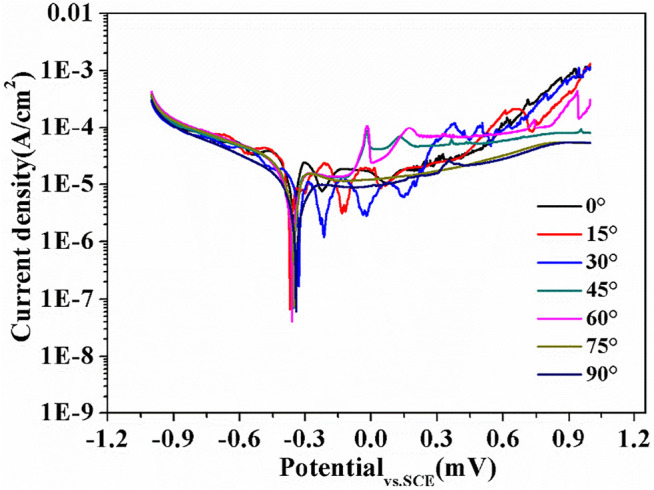
Potentiodynamic polarization curves of SLM-processed 316L SS from different angles relative to the build substrate in a 3.5 wt% NaCl solution.

Figure 10 depicts the polarization curve of all sample. The current density was not stable with the increase of sweep potential, which indicates the formation of an unstable passive film in the surface. Table 2 generalizes the sweep potential (E_corr_) and current density (I_corr_) of each sample. The sweep potential range of each sample was between −0.33 V and −0.37 V, and the 30° specimen displayed the largest sweep potential of −0.33 V. According to thermodynamics, the greater the sweep potential, the better the corrosion resistance of the sample (Zhang et al., 2012). Additionally, the current density of all samples ranged from 1.12 × 10^–5^ A/cm^2^ to 2.20 × 10^–5^ A/cm^2^, except for the 90° sample (8.8 × 10^–6^ A/cm^2^). The corrosion rate was directly proportional to the I_corr_ (Zhang et al., 2012). Therefore, the 90° sample had the lowest corrosion rate.

**TABLE 2:** Potentiodynamic polarization results of SLM-formed 316L SS.

Construction angles	I_corr_/(A/cm^2^)	E_corr_/V
0°	1.96 × 10^–5^	−0.35
15°	2.20 × 10^–5^	−0.37
30°	1.12 × 10^–5^	−0.33
45°	2.04 × 10^–5^	−0.35
60°	1.64 × 10^–5^	−0.36
75°	2.04 × 10^–5^	−0.35
90°	8.8 × 10^–6^	−0.34

## Discussions

### Effect of Angles Relative to Build Substrate on Microstructure Evolution

Reasonable process parameters can ensure that the powder is melted, and the spheroidization does not appear, which could ensure the full density of all samples, and offer high mechanical strength and corrosion resistance. Then the premature fracture or pinning corrosion would not appear due to pores during service.

The microstructure of the sample (construction angle relative to build plate) was affected due to the change of the construction angle, and the morphology of the horizontal plane was similar for each set of construction angle samples. In addition, the laser radiation and remelting of the plane of the same area increased, which caused shortened and even overlapped average laser tracks of each sample with the increase of the construction angle. When construction angle is 45°, the melt pool morphology of the vertical plane from the sample was almost the same as the laser trace of the horizontal plane. This might indicate that two samples (45° and 135° relative to the build plate) had less differences between the microstructures.

When the construction height increased, the deposition layer was continuously preheated and remelted, causing continuous accumulation of heat, while the temperature gradient and the degree of subcooling on the next layer decreased. Thus, the sub-grains nucleated and grew, leading to a larger grain size. During solidification, the growth of adjacent dendrites was inhibited due to fast cooling. Thus, columnar sub-grains were formed with obvious orientation. Portions of the columnar sub-grains exhibited epitaxial growth because of continuous remelting. During cooling, the sub-grains at the MPB continued to grow along the growth direction of the initial sub-grains inside of molten pool. The columnar grains crossed and grew further beyond the MPB. Due to the large temperature gradient during solidification, a part of the columnar grains possessed a strong epitaxial growth trend, even passing through the multilayer melt pool boundary.

The columnar sub-grains were largely different in each direction plane (relative to build plate) in SLM-fabricated 316L stainless steel. The proportion of columnar sub-grains decreased with the increase of the angle when the inclination angle of the plane was less than 45° (compared to build plate). In contrast, the proportion of columnar grains slightly increased when the inclination of the plane ranged from 45 to 60° (relative to build plate). During solidification, the heat flow dissipated through the deposited layer or the argon gas. The direction of heat transfer was parallel to the direction of grain growth, leading to competitive growth between grains (Larimian et al., 2020). Thus, the columnar grains grew upward along the BD, while the equiaxed grains appeared on the horizontal surface, but the heat transfer of each deposition layer changed with the increase in the construction angle. Based on rapid solidification, the Marangoni convection affected the heat and solute diffusion at the tip of the dendrite sub-grains, causing different aspect ratios of columnar grains in different planes (Spittle, 2013).

Nguyen et al. (2018) prepared Inconel 718 alloy with different powder layer thickness by SLM technology. When the powder layer is 20 μm, the sample exhibits a stable three-dimensional size, fine grain size, and high tensile properties. With the increase of the powder layer thickness, the porosity of the sample increases under the same process parameters. The sample exhibited partial unfused defects when the layer thickness is 50 μm. During the SLM process, when a thin powder layer is used, the sample could experience more thermal cycles. Meanwhile, the sufficient marangoni convection is conducive to a more uniform microstructure and element distribution. Compared with the thick powder layer, the columnar crystals could display the tendency of epitaxial growth in the molten pool when thin powder layer is used to build the same height, during SLM.

Figure 4 shows that <101> orientated grains decreased with the increase in the construction angle (<60°). During SLM processing, with the construction angle increases, the processing area of the block gradually decreases, that is, the area of the deposition layer decreases, which affects the liquid phase isotherm of the powder (Liu et al., 2018). During solidification, the direction of energy dissipation is affected by the building height of each sample. The growth direction of some dendrite sub-grains was perpendicular to the liquid phase isotherm, while some had a certain angle with the liquid phase isotherm. Thermal diffusivity and conductivity and element segregation led to changes of the thermal field at the melt pool. Therefore, the orientations of the grains changed. A similar phenomenon was reported in SLM-fabricated Inconel 718 (Du et al., 2019). In addition, the crystal lattice rotation due to deformation and nitriding affects texture evolution (Poulsen et al., 2003; Stinville et al., 2010). Based on the SLM process, remelting and differences in thermal fields may cause lattice rotation. Similar to the grain orientation in the build direction, the grains of different angle planes might also have undergone lattice rotation. Thus, the different planes exhibited various grain orientations.

Based on rapid melting and solidification, numerous dislocations were accumulated inside the cellular grain and columnar grain. The dislocation density gradually increased, intertwining to form dislocation walls, and further evolved into sub-grain boundaries. As the stress accumulated to a certain critical value, the sub-grain boundaries continued to absorb dislocations and then dynamic recrystallization occurred. Finally, the sub-grain boundaries gradually transformed into HAGBs (Wen et al., 2019). As the built height increased, the temperature gradient along the build direction changed greatly, increasing the density of geometrically necessary dislocation inside the sample (Mercelis and Kruth, 2006).

### Effect of Angles Relative to Build Substrate on Mechanical Properties

In this study, the yield strength (YS) of the SLM-fabricated 316 SS with different construction angles exceeded 510 MPa, much higher than that of cold-rolled 316L SS (Shin et al., 2021). This may have been related to grain refinement, solid solution strengthening, and dislocation strengthening.

During the deformation process, grain boundaries can hinder the sliding of dislocations, causing accumulation of dislocations. Therefore, the need for greater stress promoted plastic deformation and even fracture of the material. The average grain size of each sample was below 26 μm, and the volume fraction of finer grains was greater than 5% except for the 45° sample. Based on the Hall–Petch formula:

$$\Delta \sigma_{\mathrm{GB}}=\sigma_{0}+k_{y}d_{m}^{-1/2},$$

where $\sigma_{0}$ is the friction stress (≈188 MPa), k is the strength coefficient (275 MPa·μm1/2 for 316L steel), and dm represents the average grain size (Li et al., 2018; Kumar et al., 2020). For most alloys, yield strength improves with a decreasing grain size. The theoretical yield strengths of the samples due to Hall–Petch contribution were 242.8, 249.2, 245.9, and 249.6 MPa, in order of increasing construction angles (Table 3).

**TABLE 3:** Contribution of each strengthening mechanism on the yield strength of the SLM-fabricated 316L SS with different angles relative to the build substrate based on the models.

Strength (MPa)	0° sample	45° sample	60° sample	90° sample
$\Delta \sigma_{GB}$	242.8	249.2	245.9	249.6
$\Delta \sigma_{d}$	243.2	254.5	255.4	263.4
$\Delta \sigma_{ss}$	115.4	115.4	115.4	115.4
σ	601.4	619.1	616.7	628.4

Each samples had a high proportion of LAGBs and dislocation densities. During the load process, LAGBs became the nucleation center of dislocations; dislocations slipped, entangled, and formed dense dislocation walls, resulting in many secondary interfaces and leading to strain hardening. In addition, the LAGBs were related to the KAM diagram. Based on the KAM diagram, many dislocations and dislocation loops appeared in each sample. During the deformation process, the grain boundaries inhibited the movement of the dislocations, thereby leading to a high YS of the material. The dislocation strengthening is described as:

$$\Delta \sigma_{d}=\mathrm{Bb}G_{m}\sqrt{\rho },$$

where B for FCC materials is taken as 0.2, Gm is the shear modulus of the 316L steel (77 GPa), b is burgers vector, and ρ represents the dislocation density, which includes GNDs, estimated by the KAM analysis (Li et al., 2020). Based on theoretical calculations, the $\sigma_{d}$ of each sample were 243.2, 254.5, 255.4, and 263.4 MPa, in order of increasing construction angles (Table 3).

The solid solution atoms (alloying elements) dissolved in the 316L stainless steel affected yield strength. The solid solution strengthening is given by:

$$\Delta \sigma_{\mathrm{ss}}=k_{i}c_{i},$$

where ki is the strengthening coefficient and ci is the i-th alloying element’s concentration (Mohd Yusuf et al., 2020). Mohd Yusuf et al. (2020) calculated $\sigma_{ss}$ to be 115.4 MPa.

After theoretical calculation, the theoretical yield strength of 316L stainless steel can be expressed as follows:

$$\sigma =\Delta \sigma_{\mathrm{GB}}+\Delta \sigma_{d}+\Delta \sigma_{\mathrm{ss}}.$$

Grain refinement and dislocation strengthening played key roles in YS improvement of the SLM-processed 316L SS, as shown in Table 3. The results show that the theoretical strength of each sample was larger than the experimental strength. This might be attributed to the effects of defects, micro-textures, the spatial orientation of the melt pool boundary, and element segregation. Among them, the 90° sample contained large pore, which affects the yield strength of the sample.

In addition, the plasticity of the SLM-processed 316L SS was greater than 45% in this study. Some researchers consider that the high ductility of SLM-prepared 316L SS is owed to the interaction between deformation twins and dislocations in the unit cell. The strain-induced texture evolution played a character in improving the ductility of SLM-processed 316L SS before deformation (Dryepondt et al., 2021). Pham et al. (2017) found that the twinning activity was very active near the necking area, and the slip could re-orient the twinning direction. During deformation, the microstructure of the 316L SS underwent grain rotation, slipping, and twinning (Liu et al., 2020). The dispersion of the refined grain structure could improve the strength and ductility of the alloy. In addition, the association of both fine grains and coarse grains was beneficial to improve the elongation (Agius et al., 2017). All the samples exhibited a combination of fine grains and coarse grains, except for the 45° sample, which ensured the high elongation of the SLM-processed 316L SS. During the deformation process, a greater accumulation of dislocations occurred near the grain boundaries in the 90° sample due to the differences in grain sizes. Meanwhile, based on the rough-induced crack termination mechanism, friction stress occurred and slowed the propagation of cracks in the 90° sample, thus the 90° sample had higher ductility than the other samples (Koyama et al., 2017).

The strength and toughness of each set of construction angle samples (for which the sum of the construction angles was 90°) seemed to be similar (within the error of 20 MPa or 3%, respectively), except for the 0° and 90° specimens. Each set of construction angle samples (for which the sum of construction angles = 90°) showed similar macrostructure on the vertical plane and the horizontal plane. This shows that the SLM-processed 316L SS had similar macrostructures on planes with different angles, for which the sum of the construction angles = 90° (relative to build plate). During SLM, the laser acted on the powder layer, melting the metal powder, which solidified and formed a good metallurgical bond. The horizontal plane of each sample shows the morphology of “track–track,” and the vertical plane of each sample shows the morphology of “layer–layer,” which is the typical macrostructure morphology of SLM-processed samples. Based on rapid cooling, the SLM-processed samples had a fine microstructure and few element segregations at the grain boundaries. Thus, the 316L SS showed strength and plasticity comparable to forging.

### Effect of Angles Relative to Build Substrate on Corrosion Resistance

During corrosion, the oxide bonding of the passive film became weak and dissolved when Cl^−^ ions attacked through the pores of the 316L SS sample surface. The passive film could rupture, thereby affecting the corrosion properties. In addition, based on ASTM G102, the corrosion rate of each sample is calculated by Faraday’s law, and the mass loss rate (MR) is calculated as follow:

$$\mathrm{MR}=K_{2}i_{\mathrm{corr}}\mathrm{EW,}$$

where MR is given in g/m^2^d, i_corr_ in A/m^2^, K_2_ is a constant (0.8953 g/Ad),  ρ is the density of materials, and EW may be thought of as the mass of metal, which may be oxidized. According to the i_corr_ in Table 2, the mass loss rate of each sample is different. It can be related to the pore, dislocation density, grain size, and microstructure.

The few of pores had an important role in improving corrosion performance (Mohd Yusuf et al., 2018). The metastable pits were more easily transformed into stable pits due to grain refinement. Therefore, the austenitic stainless steel was hardly affected by corrosion (Abbasi Aghuy et al., 2015). Based on rapid melting and solidification, manganese sulfide (MnS) inclusions were eliminated or significantly refined in the SLM-formed 316L SS, which improved the corrosion resistance (Laleh et al., 2019a).

The higher the proportion of LAGBs in the sample, the stronger the corrosion resistance due to the low interface energy of LAGBs (Laleh et al., 2019b). The greater dislocation density in SLM-formed 316L SS provided further nucleation sites for the passive film (Wang et al., 2015). The increased grain boundary density was conducive to enhancing the formation and adhesion of the passive film on the sample surface (Al-Mamun et al., 2020). The performance of the passive film directly affected the corrosion resistance of each sample. The passive film of 316L SS is made up of Fe oxide on the outer layer and Cr oxide on the inside layer, which has semiconductor properties (Kong et al., 2018b). When the semiconductor was immersed in the NaCl solution, a Helmholtz layer formed on the solution side while a space–charge layer formed on the semiconductor side. The surface of the p-type semiconductor electrode (Cr oxide) had a negative residual charge, and the Helmholtz layer was a cationic layer, which prevented the adsorption of Cl on the sample and thus the passive film’s erosion (Hatakeyama et al., 2020). Hence, the 90° sample had high LAGBs, dislocation density, and finer grains, thus it exhibited good corrosion resistance.

## Conclusion

In this study, the effects of construction angles (from 0° to 90° relative to build plate) on the microstructure evolution, texture, tensile properties, and corrosion resistance of SLM-prepared 316L samples were researched. The main conclusions are made as follows:

1) With the increase of angle, “track–track” overlap track showed various variations on the horizontal surface. Each set of construction angle samples (for which the sum of construction angles = 90°) exhibited shortening and even overlapping of the laser track.
2) Increasing the angle relative to the build substrate gradually changed the grain morphology of the 316L SS from columnar grains to equiaxed grains. The 0° sample and 90° sample showed a strong <101 > texture. The grain orientation showed a trend from <101> to <111> with an increasing construction angle (<60°).
3) A good unity of high strength (UTS = 708 MPa, YS = 588 MPa) and ductility (54.51%) exhibited in the 60° sample.
4) The 90° sample showed higher elongation (62.57%) and corrosion resistance than the other samples. The passive film had more nucleation sites and formed on the sample surface, by reason of the higher dislocation density and a refined grain structure.

## Data Availability

The raw data supporting the conclusions of this article will be made available by the authors, without undue reservation.
